# Challenges in computational fluid dynamics applications for bone tissue engineering

**DOI:** 10.1098/rspa.2021.0607

**Published:** 2022-01

**Authors:** Tiago Pires, John W. C. Dunlop, Paulo Rui Fernandes, André P. G. Castro

**Affiliations:** ^1^ IDMEC, Instituto Superior Técnico, Universidade de Lisboa, Lisboa, Portugal; ^2^ MorphoPhysics Group, Department of the Chemistry and Physics of Materials, University of Salzburg, Salzburg, Austria

**Keywords:** computational fluid dynamics, bone tissue engineering, optimization, scaffolds, biomechanics

## Abstract

Bone injuries or defects that require invasive surgical treatment are a serious clinical issue, particularly when it comes to treatment success and effectiveness. Accordingly, bone tissue engineering (BTE) has been researching the use of computational fluid dynamics (CFD) analysis tools to assist in designing optimal scaffolds that better promote bone growth and repair. This paper aims to offer a comprehensive review of recent studies that use CFD analysis in BTE. The mechanical and fluidic properties of a given scaffold are coupled to each other via the scaffold architecture, meaning an optimization of one may negatively affect the other. For example, designs that improve scaffold permeability normally result in a decreased average wall shear stress. Linked with these findings, it appears there are very few studies in this area that state a specific application for their scaffolds and those that do are focused on *in vitro* bioreactor environments. Finally, this review also demonstrates a scarcity of studies that combine CFD with optimization methods to improve scaffold design. This highlights an important direction of research for the development of the next generation of BTE scaffolds.

## 1. Introduction

Bones are some of the most important tissues in the human body, being responsible for providing structural support, protecting important internal organs and maintaining mineral homeostasis. Therefore, a quick intervention is vital to treat any injury or defect on this tissue that cannot be easily repaired. A common treatment method for bone injuries or defects is bone grafting [1]. However, this treatment presents considerable drawbacks, such as donor site morbidities and higher risk of infections. Taking these limitations into consideration, bone tissue engineering (BTE) has been looking into the use of scaffolds as an appealing alternative for the treatment of bone injuries and bone defects.

**Figure 1.**
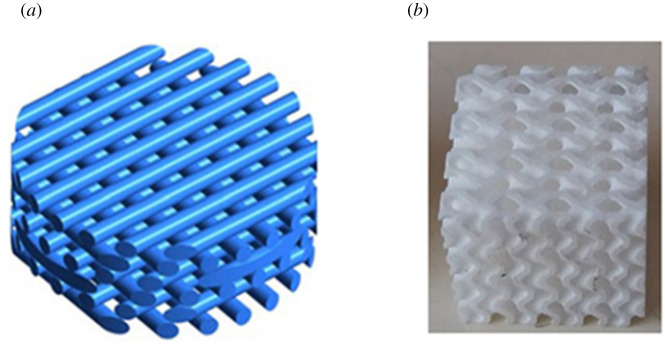
Examples of possible scaffold geometries for bone tissue engineering (BTE): (*a*) lattice geometry (adapted from [5]) and (*b*) triply periodic minimum surfaces (TPMS) [6]. (Online version in colour.)

Scaffolds are porous support matrixes designed to allow cell growth, while maintaining the mechanical properties inherent to bone tissue. These structures can be random porous solids [2], but can also be designed to have targeted geometries, such as simple lattices [3] or more complex structures such as the triply periodic minimum surface (TPMS) approach [4] (figure 1). To promote cellular growth, scaffolds must account for the mass-transport requirements of cell nutrition and for the interconnectivity of porous channels for cell migration and surface conditions for cell attachment [7]. One parameter that is usually studied regarding these requirements is the scaffold's permeability. This is a fundamental characteristic of any BTE scaffold, because higher permeability facilitates cells entering the scaffold as well as easing the distribution of nutrients through the scaffold. These factors, in turn, translate to more favourable conditions for cellular growth. Another important parameter to study during a scaffold's design is wall shear stress (WSS) that affects the cells inside the scaffold. WSS arises through load-driven fluid flow from relative movement between the scaffold and the cell and tissue containing fluidic phase within the scaffold. Studies have shown that different levels of WSS result in different mechanical signals affecting the mesenchymal stromal cells, resulting in differences to the cellular differentiation process [8,9].

Studies have shown that, during the scaffold design process, small changes to the pore size and/or porosity might considerably influence the scaffold's mechanical support and the process of cell growth and tissue infiltration [10,11]. Other factors that might also influence scaffold properties are the materials and manufacturing techniques that are chosen [12,13]. These multiple inputs for scaffold development result in a large range of possible designs: in the conceptualization of a new scaffold, one fundamental step is to ascertain its properties through computational simulations, to save time and manufacturing/prototyping costs.

Computational methods have been used in various engineering fields to reduce costs and optimize the desired characteristics [14]. This is especially true in BTE, where computational simulations allow scaffolds to be designed and tested prior to manufacturing, thus reducing the costs associated with creating new scaffolds. Furthermore, numerical methods also permit the testing of a much larger range of scaffold designs by allowing changes to a scaffold material and simple geometric properties (such as the wall thickness), without the need to re-manufacture the entire structure. One of the most important types of simulations in BTE is computational fluid dynamics (CFD), which allows the study of the fluid passing through the scaffold (more specifically, the study of the permeability, fluid velocity and WSS), permitting a better understanding of how each scaffold geometry influences the cell growth process [15,16].

Additionally, computational simulations can also be used to design the scaffold geometry using an optimization approach (by attempting to optimize scaffold characteristics such as their Young's modulus [17]; compressive strength [18] or octahedral shear strain [19–21]), instead of merely analysing the properties of the scaffolds. However, optimization strategies in BTE have almost exclusively focused on the mechanical properties of the scaffold itself, disregarding the fluid flow inside the structure, and consequently, the interaction between the cells and the scaffolds.

Taking into consideration the importance of CFD analyses and optimization techniques for BTE, this review attempts to identify the current limitations facing the use of CFD in scaffold design. Additionally, this review also highlights some possible alternatives that could help a new generation of BTE scaffolds that overcomes said limitations. Therefore, this paper provides a comprehensive view of publications from 2015 onwards that used CFD analyses to study BTE scaffolds or played a role in designing said scaffolds. This review first delves into the general framework of most CFD analyses, followed by an overview on recent applications of CFD in BTE. Then we present recent studies that combine CFD with other techniques such as the finite-element method (FEM), experimental validation or scaffold design optimization. Finally, we discuss the current limitations of the field and enumerate the challenges of integrating CFD with scaffold design optimization.

## 2. CFD

CFD is a computational approach to modelling the fluid flow of a certain fluid domain by numerically solving the Navier–Stokes equations. This method can be implemented by using several distinct techniques; however, the approach chosen for a majority of CFD analyses is the finite volume method (FVM). The FVM consists in a numerical method that attempts to resolve the conservation laws by applying them over differential volumes and finding the solution for the resulting equations [22]. The FVM presents two advantages over alternative methods for solving numerical fluid simulations: it is strictly conservative, and it has an easier implementation of boundary conditions. In flow dynamics, the flux that exits from a given volume face must be equal to the flux entering the adjacent face and because the FVM is based on volumes instead of elements, mass, energy and momentum remain conserved locally, making it preferable over other methods, such as the FEM. Furthermore, since all of the unknown variables are evaluated at the centroid of the volume, it is less invasive to insert boundary constraints in the FVM compared to other methods.

When analysing scaffolds meant for BTE, CFD studies usually consist of a fluid flow starting at a given inlet surface(s), passing through the scaffold and exiting at an outlet surface(s). The fluid is normally modelled as a Newtonian fluid with constant density and viscosity (in most cases the fluid is assumed to be water); the inlets are velocity inlets with a constant velocity and the outlets are pressure outlets set to 0 Pa. Although this is the most commonly used configuration, some papers have conducted analysis with different conditions, such as the simulation of non-Newtonian fluids [23] and non-constant inlet flow rates [24,25].

**Figure 2.**
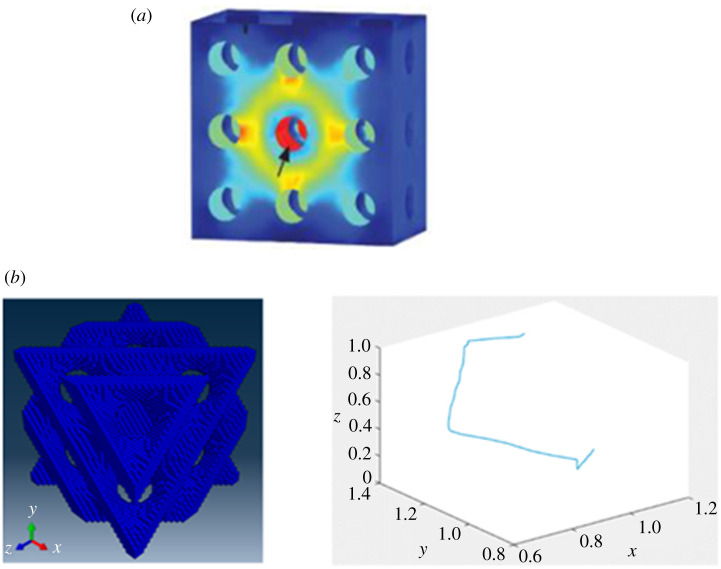
Fluidic properties studied using CFD simulations: (*a*) wall shear stress (WSS) along the walls of the scaffold (adapted from [26]) and (*b*) tortuosity of the fluid flow through the scaffold (adapted from [27]). (Online version in colour.)

CFD simulations are normally used to study specific properties of scaffolds that are related to the fluid flow, with almost all CFD studies analysing the permeability and most of them also analysing the WSS (figure 2*a*) [23,26,28–36]. Additionally, these analyses are sometimes used to study the tortuosity of the fluid flow [6,27,37] (figure 2*b*) or to examine the possible cellular distribution inside the scaffolds [24,25]. Alternatively, CFD simulations have also been employed by a couple of studies for considerably distinct applications. These include the study by Rouhollahi *et al*. [38] who used this computational method to determine the average pore size and pore distribution in Freeze-Cast scaffolds and the paper by Chappard *et al*. [37] who used CFD to determine the permeability of different granule biomaterials for mandible scaffolds.

## 3. Current applications of CFD in BTE

In recent years, several studies have been conducted that implemented CFD analyses on scaffolds meant for BTE. Most of these papers normally focus on either analysing fluidic properties of the structures or analysing the cellular behaviour inside the scaffolds (table 1).

**Table 1.** Review of CFD studies, grouped by objectives.

objectives	analysed parameters	reference	application	material	geometry
fluidic properties	permeability; WSS	[23]	generic	non-applicable	lattice/struts; gyroids
	permeability; WSS	[28]	generic	non-applicable	square pores
	permeability; WSS	[30]	generic	non-applicable	TPMS; lattice/struts
	permeability; WSS	[26]	generic	magnesium	circular pores
	WSS	[39]	generic	polyamide EOSINT P/PA2200	circular pores
	fluid velocity; WSS	[5]	generic	PCL	lattice/struts (µCT scan)
	fluid velocity; WSS	[40]	bioreactor	non-applicable	granular packings saffolds
	WSS; fluid velocity; pressure	[41]	generic	β-TCP (β-tricalcium phosphate)	lattice/struts; freeze-drying; dog femur
	permeability; fluid velocity; fluid streamlines	[42]	generic	stainless steel	gyroid
	permeability; WSS	[31]	generic	HA–PMMA	µCT of manufactured scaffolds
	permeability	[43]	generic	non-applicable	TPMS; lattice/struts
	permeability; flow rate; fluid shear stress	[44]	generic	Ti–6Al–4 V	lattice/struts
	permeability	[45]	generic	non-applicable	circular pores
	permeability	[46]	generic	non-applicable	circular Pores
	pressure; mass flow; WSS	[32]	generic	unspecified biodegradable organic polymer	TPMS
	WSS; fluid velocity	[33]	generic	poly(d,l-lactide) (PDLLA)	rectangular/circular pores
	permeability; WSS	[34]	bioreactor	β-tricalcium phosphate (β-TCP)	cylindrical scaffold with spherical pores
	permeability	[47]	generic	black photopolymer resin	TPMS
fluidic and mechanical properties	permeability; WSS; compressive strength; Young's modulus	[48]	generic	Ti–6Al–4 V	gyroid; lattice
	compressive strength; Young's modulus; permeability; stress concentration	[49]	generic	Ti–6Al–4 V	lattice/struts
	maximum compressive stress; Young's modulus; permeability	[50]	generic	Ti–6Al–4 V	Voronoi method; lattice/struts
	Young's modulus; shear modulus; permeability	[51]	spinal interbody fusion cage	undefined	lattice/struts
	Young's modulus; permeability	[52]	generic	poly(d, l-lactide) (PDLLA)	Voronoi method
	mechanical strength; structural modulus; fluid shear stress	[53]	generic	P430 ABS	custom
	Young's modulus; compressive strength; yield strain; permeability	[54]	generic	calcium sulfate	TPMS
	Young's modulus; yield strength; permeability; WSS	[55]	generic	Fe	circular pores
	permeability; Young's modulus; compressive strength	[56]	generic	Ti–6Al–4 V	lattice/struts; gyroids; Schwarz primitive
	fluid velocity; WSS; compressive strain	[57]	generic	poly(d, l-lactide) (PDLLA)	lattice/struts (µCT scan)
	Young's modulus; fluid velocity; axial strain; permeability; WSS	[58]	generic	TiO_2_	foam
cell growth analysis	shear stress	[59]	bioreactor	Ti–6Al–4 V	custom
	shear strain; shear stress	[60]	generic	block copolymer 300PEOT55PBT45 (PolyVation B.V.)	lattice/struts
	WSS	[35]	bioreactor	non-applicable	rectangular/circular pores
	WSS	[36]	generic	non-applicable	lattice/struts
predicting cell migration	fluid streamlines; cell position	[24]	bioreactor	PCL	lattice/struts
	fluid streamlines; cell position	[25]	bioreactor	PCL	lattice/struts
optimization	compressive strength; permeability	[61]	generic	ceramic	lattice/struts
new computational technique for irregular pores	WSS	[29]	bioreactor	silk fibroin (SF)	micro-CT

### (a) Analysing fluidic properties

Tissue engineering scaffolds are essentially designed with the objective of promoting cell growth [62]. Therefore, whether a scaffold is meant for *in vitro* or *in vivo* applications, it must possess the required characteristics to promote the desired rates of cellular differentiation and growth. These characteristics can be determined by examining the fluid flow passing through the scaffold, normally by employing a CFD analysis. As previously mentioned, the two main parameters that are studied are the scaffolds' permeability and WSS.

Permeability has been demonstrated to be essential for cellular growth, with more permeable scaffolds generating more favourable conditions [10]. However, it should be highlighted that high permeability scaffolds also present some limitations, namely, lower overall mechanical properties and the reduction of the cell–scaffold interaction [11]. This is because high permeability is normally caused by high porosity, which might not allow sufficiently long entrapment of the cells inside the scaffold. Such limitation would cause them to not adhere to the scaffold wall [15]. Singh *et al*. [46] used a CFD analysis to determine the permeability of various scaffolds with circular pores. They discovered that their structures presented permeabilities in the range of natural bone when the pores had a diameter between 0.5 and 1.5 mm. Rahbari *et al*. [45] also evaluated the permeability of cylindrical pored scaffolds and determined their permeability coefficient. They found that, contrary to past research, the trend of the variation of pressure drop with mass flow rate was exponential instead of linear. Furthermore, the study also found that at higher porosities, the pore shape had a considerable higher impact on the scaffold permeability, with hollow structures being much more permeable than tubular structures.

Besides permeability, the work of Prendergast *et al*. (and subsequent studies) discussed how a combination of the velocity of the fluid flow and the shear stress influences the cellular differentiation process (denominated as ‘mechanobiological output’), underlining the importance of both of these parameters [11,63–65]. Begum & Arumaikkannu [39] analysed the WSS of 15 customized scaffolds with circular pores. The study found, as expected, that the scaffold geometries with the smaller pore sizes resulted in the highest values of WSS. Ouyang *et al*. [44] conducted an experimental study to evaluate how changes to a porous lattice scaffold geometry affects cellular response and bone regeneration. Alongside an experimental component, the study also used a numerical CFD analysis to better evaluate the permeability of the scaffolds as well as the WSS and fluid velocity along the structure. The results suggested that the scaffolds with larger pores were preferable for cell penetration, while scaffolds with smaller pores were more conducive to cell deposition.

When studying the WSS of a given structure, a problem arises for irregular geometries, given the high computational cost of running a CFD analysis on the whole structure. To overcome this, Zhao *et al*. [29] developed a multi-scale CFD approach to quantify the micro-fluidic environment in irregular scaffold geometries. The multi-scale framework consists of a macro-model of the entire scaffold and a detailed micro-model of a representative portion of the scaffold. This new approach was successfully validated using a silk fibroin scaffold.

Alongside the CFD analysis, a number of studies also implement a fluid structure interaction (FSI) analysis [26,33,34]. This method is used to obtain a better understanding of how differences in scaffolds (such as different geometries or pore sizes) influence the shear stress on scaffold surfaces and consequently the cell adhering to those surfaces. Zhao *et al*. [33,34] used CFD alongside FSI to investigate how the geometry of scaffolds, with rectangular or circular pores, influences the WSS of the structure. They discovered that combined stimuli (fluid perfusion and mechanical compression) caused an amplified WSS, instead of a simple superposition for each isolated system. FSI was also used to examine how interstitial cell formation influenced the WSS of the scaffold. It was found that after 28 days of cellular growth, the permeability of the scaffold was approximately a 10th of its original value, with tissue growing within the pores rather than on its struts. In terms of the WSS, this was dependent on three factors: (i) the volume of the present interstitial tissue; (ii) the morphology of said tissue; and (iii) the location of the tissue in the scaffold. Nevertheless, it was shown that interstitial tissue could lead to a 10 times increase in the WSS affecting the cells: in order to maintain a proper cellular stimulation, the flow rate might need to be adjusted during growth. Basri *et al*. [26] investigated effects of degradation on magnesium scaffolds with circular pores, evaluating their permeability and WSS before and after the degradation. The areas with higher WSS had a considerably greater material degradation.

As the previous studies have demonstrated, the design parameter of a given scaffold that most influences the fluidic properties is its geometry. Accordingly, many studies have used CFD to investigate various scaffold geometries. Lin *et al*. [41] used CFD to investigate the fluid velocity, pressure and WSS of three distinct scaffold geometries: artificially designed lattice scaffolds, a scaffold produced using freeze-drying and the geometry derived from a dog femur as a reference criterion. These latter two geometries were obtained through micro-computed tomography (µCT). The researchers discovered that the designed scaffold showed much closer values of WSS to the reference geometry than the freeze-drying scaffold. Cruel *et al*. [40] investigated WSS levels on different scaffold geometries produced via random granular packings. They tested three different geometries, which were the Ccube, C1bead and C2bead, each named after the particle that was used to design them (3 mm sided cubes, 2 mm diameter beads and 3.5 mm diameter beads, respectively). The results demonstrated how these new designs could present a possible alternative to existing geometries. Out of the three tested geometries, the C2bead configuration was the best in terms of WSS levels, distribution and homogeneity.

**Figure 3.**
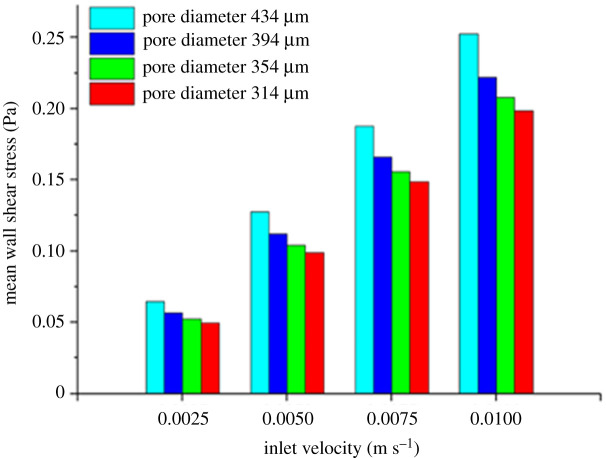
Average WSS in scaffolds with different pore diameters and fluid inlet velocities (adapted from [32]). (Online version in colour.)

A promising scaffold geometry that has also been the focus of recent studies is the TPMS. A minimal curve surface is defined as a surface that is locally area-minimizing, meaning that for a given boundary condition, these surfaces have a minimal surface area. The geometries are also symmetric in three independent directions, thus making them triply periodic. Montazerian *et al*. [43] studied the longitudinal and radial permeability of TPMS and lattice scaffolds. They concluded that TPMS scaffolds, especially the I-WP geometry, are overall more permeable than lattice scaffolds. They also discovered that radial permeability appears to be a more accurate indicator of cell growth behaviour than the conventional longitudinal permeability. Ali *et al*. [30] also compare lattice with TPMS scaffolds, but focus on their permeability as well as their WSS. Out of the eight tested geometries, the results showed that the lattice-diamond structures presented the highest permeability and one of the highest WSS, making it the preferable geometry for BTE. Furthermore, the study also reported that it was not possible to find a direct correlation between the architecture of the scaffold and its WSS distribution, highlighting the complexity in designing a scaffold with an optimal WSS distribution. Ma *et al*. [42] and Wang *et al*. [32] used experimental and numerical methods to examine the properties of TPMS scaffolds (figure 3). The CFD analyses demonstrated how the tested structures revealed favourable permeability and fluid streamlines and WSS that could promote cell seeding efficiency and cellular growth. Zhianmanesh *et al*. [47] analysed the permeability of cylindrical TPMS scaffolds with radially graded porosity. Their study indicates that although both the central and peripheral porosity have a considerable impact on structural permeability, the permeability is much more dependent on the peripheral porosity. Furthermore, the paper also highlights that while some geometries have the highest permeability for non-uniform porosities (I-WP, G and Fxyz-Fxxx2), this is not true for all of them, as one specific geometry (IJ*-P2) has the highest permeability for a uniform porosity.

When designing BTE scaffolds, two related characteristics that also influence the viability of a given scaffold, besides its geometry, are the chosen material and the manufacturing process. Although a scaffold's material does not directly affect its fluidic properties, it can indirectly affect WSS and permeability, because different materials are associated with different manufacturing techniques. Furthermore, a scaffold manufacturing process greatly influences its properties, because different manufacturing techniques possess distinct levels of accuracy. Accordingly, many studies have implemented computational methods to evaluate how differences in materials and manufacturing alter the fluidic properties of the scaffold. Mahammod *et al*. [31] used a CFD analysis alongside mechanical compression tests to investigate the properties of different hydroxypatite–polymethylmethacrylate (HA/PMMA) composite scaffolds. The CFD analysis was conducted on geometries obtained through µCT of composite scaffolds fabricated with varying weight percentages of HA in a PMMA matrix (50–70%). The results showed that an increase in the scaffolds' porosity leads to an increase in their permeability, but also results in lower WSS (figure 3). Taking this into consideration, alongside the results of the compression test, leads to the conclusion that the composite scaffold with 60% HA content is the preferable option for BTE applications. Campos Marin & Lacroix [5] investigated the WSS and fluid velocity on five supposedly identical scaffolds, created through a rapid prototypes technique. µCT analyses were conducted on each of the five polycaprolactone (PCL) scaffolds and the resulting geometries were studied using CFD. The results revealed that there was a considerable variability between the WSS and fluid velocities of the ideal initial scaffold geometry and the five manufactured geometries. The researchers argue that this variability is inherent to all rapid prototyping manufactured scaffolds, requiring a more systematic analysis of such scaffolds in any pre-clinical and clinical tests.

Finally, some recent studies have also investigated how other factors influence the fluidic properties of a given scaffold. Ali & Sen [23] used CFD analyses to compare the permeability and WSS of lattice scaffolds with Newtonian and non-Newtonian fluid models. This study was conducted because most numerical models simplify blood, which is a non-Newtonian fluid, into a Newtonian fluid. The study presents a clear distinction between the models, with the non-Newtonian model revealing considerably lower permeability and overall higher WSS. These results underline the need for further numerical BTE scaffolds studies that incorporate a non-Newtonian blood model. They also studied the role of surface roughness on the permeability and WSS [28]. The study found that the effects of surface roughness on permeability were negligible when compared with the effects of pore sizes. Furthermore, surface roughness had the biggest impact on permeability on the scaffolds with the smallest pore sizes (300 µm). However, the effects of this parameter were much more significant on the WSS. On scaffolds with larger pore sizes, rough surfaces created more favourable conditions for cell attachment. On scaffolds with smaller pores, rough surfaces had the opposite effect, causing channel occlusion, which inhibited cell differentiation and proliferation.

### (b) Analysing cellular behaviour

Computational simulations are essential to analyse the mechanical and fluidic properties of a given scaffold. However, these methods are not limited to merely determining these characteristics. Several studies have used CFD simulations in conjunction with cellular models to evaluate how different scaffolds influence cell growth [59] and cell differentiation [60].

Guyot *et al*. [59] used a CFD analysis alongside a previously developed growth model [66,67] to predict the cellular growth in a titanium scaffold meant for a perfusion bioreactor. This study considered the following equation to study the cells behaviour:

3.1 $$V_{G}=A*g(k)*f(\text{S}{\text{S}}_{\text{surf}}),$$

where *V_G_* is the local neotissue growth velocity, *A* is the neotissue growth velocity parameter (determined experimentally), *g*(*k*) is the mean curvature influence function (with *k* being the local mean curvature) (equation (3.2)) and *f*(SS_surf_) is the surface shear stress influence function [66] (equation (3.3)):

3.2 $$g(k)=\left\{ \begin{array}{ll} -k & \text{if}\;k>0\\ 0 & \text{if}\;k\leq 0, \end{array}\right.$$

and

3.3 $$f(\text{S}{\text{S}}_{\text{surf}})=\left\{ \begin{array}{ll} 0.5+\frac{0.5*\text{S}{\text{S}}_{\text{surf}}}{a_{1}} & 0\leq \text{S}{\text{S}}_{\text{surf}}<a_{1}\\ 1 & a_{1}\leq \text{S}{\text{S}}_{\text{surf}}<a_{2}\\ \frac{\text{S}{\text{S}}_{\text{surf}}-a_{3}}{a_{2}-a_{3}} & a_{2}\leq \text{S}{\text{S}}_{\text{surf}}<a_{3}\\ 0 & a_{3}\leq \text{S}{\text{S}}_{\text{surf}} \end{array}\right.$$

**Figure 4.**
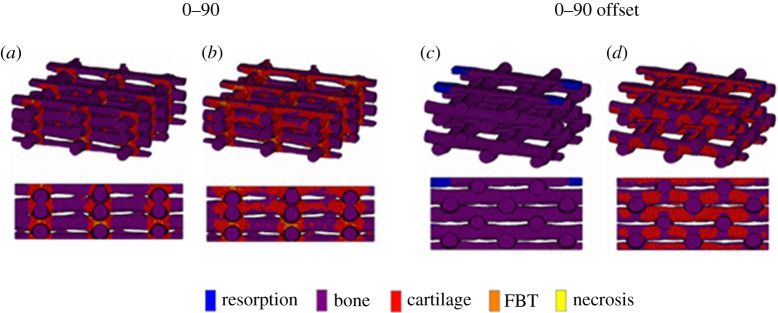
Surface shear strain of: (*a*) a 0–90 scaffold with no flow; (*b*) a 0–90 scaffold with flow; (*c*) a 0–90 offset scaffold with no flow; and (*d*) a 0–90 offset scaffold with flow (adapted from [60]). (Online version in colour.)

The implemented model predicts the development of local neotissue based on the curvature of the surface and the shear stress induced by the fluid flow. The authors find that there is a lower amount of neotissue formation at the periphery of the scaffolds because of the lower flow-induced shear stress at that location. The study also reports that when different fluid flow rates are applied, a considerable distinction in shear stress is noted throughout the scaffolds, with the lower flow rates causing lower shear stresses, resulting in lower cell growth. Hendrikson *et al*. [60] used a combination of CFD and the FEM to study how different lattice scaffold geometries influence shear stresses and shear strains inside the structures. Their results were then coupled with Prendergast mechano-regulation theory [64], to evaluate how the distinct geometries translate to differences in cell differentiation (figure 4). The study demonstrated a clear correlation between the geometry of a scaffold and the cell differentiation process, with a higher overall bone formation on their 0–90 offset geometry.

Zhao *et al*. [35] also used CFD analysis to study the WSS and consequently the behaviour of cells inside different scaffolds with either rectangular or circular pores. This study was conducted to determine the optimal flow rate to induce mineralization for a perfusion bioreactor. The study concluded that the optimal flow rate corresponded to the range between 0.5 and 5 ml min^−1^ (which corresponds to fluid velocities between 0.166 and 1.66 mm s^−1^), with lower flow rates not inducing mineralization and higher flow rates causing cellular death. Another study also investigated the effect of flow rate on the WSS inside the scaffolds [36]. That paper focused on evaluating whether a time-varying flow rate was required to maintain the WSS in the optimal range. They found that, because of the formation and growth of the extracellular matrix (ECM) over time, a linear reduction of the flow rate was preferable because it resulted in a higher percentage of the ECM surface to be exposed to the optimal WSS after cellular growth.

Besides cellular growth and differentiation, a couple of studies have also used CFD analyses to better understand the positions of cells inside a scaffold at a given time. Campos Marin *et al*. [24] studied the positions of cells inside a scaffold, in bioreactor conditions, to determine which were the most relevant factors in the occurrence of cell deposition. They found that the only two mechanisms that drive cells towards the scaffolds walls are secondary flows and the effect of gravity. Further research into the effect of gravity [25] revealed that its impact is very dependent on the fluid flow rate. Low flow rates result in a poor transport of cells to the scaffold, while high flow rates overcome the effect of gravity but also minimize the interaction between the cells and the scaffold walls, causing the cells to pass through the structure.

## 4. Beyond CFD

Scaffold design is a complex process that involves more aspects that simply determining the fluidic properties of different geometries and how they influence cellular behaviour. Taking this into consideration, this section looks into how CFD simulations can be used alongside other techniques during scaffold design, more specifically FEM analysis, experimental validation and optimization processes.

### (a) FEM analysis alongside CFD

Scaffolds that are meant to replace injured bone tissue need to possess the required mechanical properties to maintain the balance of providing adequate mechanical support to the developing bone tissue [7] while avoiding the occurrence of stress shielding of the surrounding bone [68]. An exception to this requirement are scaffolds focused on *in vitro* applications such as bioreactors [24,25,29,34,35,40,59]. This is because these applications are more focused on improving cellular differentiation and growth, thus only need to maintain enough mechanical support to allow cellular attachment.

CFD only analyses the behaviour of the fluid within a scaffold and the mechanical properties of the actual scaffold are commonly estimated using the computational method of FEM. This method attempts to solve a chosen set of mathematical equations of a given domain by first dividing it into smaller subdomains (referred to as finite-elements) and then solving the equations for each subdomain. These subsolutions are then pieced together to solve over the entire domain.

**Figure 5.**
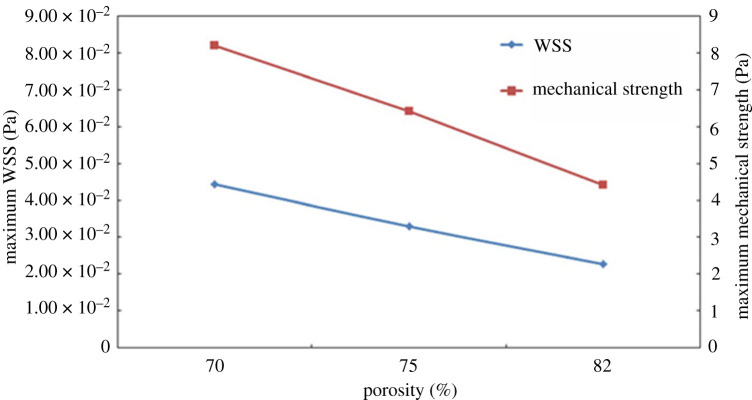
Maximum mechanical stress and maximum WSS in function of the scaffold porosity (adapted from [53]). (Online version in colour.)

Different computational studies have given emphasis to distinct mechanical properties, all of which are important in creating a functional BTE scaffold. The two mechanical parameters of a scaffold that are regularly examined are its compressive strength [48,49,54,56] and Young's modulus [48–52,54–56,58]. Any scaffold meant for an *in vivo* application needs to have an appropriate compressive strength and Young's modulus providing the required mechanical support to the implantation site, without resulting in a large loss of bone mass due to stress shielding. Kantaros *et al.* [53] and Arjunan *et al*. [49] investigated both the fluidic as well as the mechanical properties of simple lattice scaffolds. These studies found that the mechanical properties of the scaffold were the best at the lowest porosity. Additionally, Kantaros *et al*. [53] also found that the WSS, similar to the mechanical properties, was better at lower porosities (figure 5). Noordin *et al*. [55] also performed similar studies on various Fe scaffolds with circular interconnected 800 µm pores of varying porosities. They also concluded that lower porosity resulted in better mechanical properties and WSS, but at the cost of a lower permeability (as discussed in the previous section). Egan *et al*. [51] tested various lattice-based structures for a spinal interbody fusion cage application. This study determined that the cube lattice topology resulted in the highest Young's modulus of all of the scaffolds while maintaining a high permeability, but it also presented the lowest shear modulus. Conversely, the octet topology resulted in overall high shear modulus but low permeability, meaning it could be useful for cellular growth conditions which are not limited by nutrient transport.

As previously discussed, TPMS geometries are an appealing choice for scaffold design, and several numerical studies simultaneously focused on their fluidic and mechanical properties. Montazerian *et al*. [54] used numerical methods to evaluate the fluidic and mechanical properties of 240 different TPMS geometries. They divided the geometries into two groups: high strength structures and low stiffness structures. The study concluded that, for 70% porosity, two of the analysed structures (P* and Ixxx-J*) had the highest overall Young's modulus, compressive strength and permeability. For the analysed low stiffness structures, a specific scaffold (Fxyz-Fxxx2) had the strongest and most permeable geometry. The paper also presented the possibility of combining the two groups of structures to place the high strength geometries in the load-bearing regions while allowing the low stiffness geometries to be in the most biological significant region. Ali & Sen [48] and Yu *et al*. [56] both studied the properties of titanium lattice and TPMS scaffolds using CFD and the FEM. Ali & Sen [48] concluded that the lattice geometries had a higher compressive strength and permeability than the equivalent gyroid geometry. Nevertheless, the latter design (with an 80% porosity) had the closest permeability, WSS distribution and Young's modulus to human bone, making it the best choice for a BTE scaffold. Yu *et al*. [56] also concluded that the lattice geometries presented the highest permeabilities. However, contrary to the previous paper, they concluded that the gyroid scaffolds had a much higher compressive strength than the lattice scaffolds.

Besides the TPMS geometry, a couple of studies have also been conducted to evaluate scaffolds created by using the Voronoi tessellation method. Du *et al*. [50] tested the properties of lattice scaffolds as well as scaffolds created by the Voronoi tessellation method. As expected, the study found that higher porosity led to an increase in the scaffolds' permeability, at the expense of their compressive strength. They also concluded that the Voronoi tessellation method resulted in irregular porous structures that more closely imitated the human bone structure. Gómez *et al*. [52] studied structures created by this method and also found that the resulting scaffolds could have applications in BTE. They discussed how the properties of the scaffolds are directly dependent on structural characteristics, such as porosity, trabecular thickness, trabecular separation and trabecular number.

The combination of the FEM and CFD analysis has also seen some use in other areas of study in BTE. Zhang *et al*. [58] used these computational methods to compare new titanium dioxide (TiO_2_) scaffolds with existing, commercial materials (Bio-Oss, Cerabone and Maxresorb). The results showed that the TiO_2_ had a better permeability and WSS distribution than the existing commercial materials but had a lower Young's modulus. Similar to the studies that also used FSI discussed in the previous section, Zhao *et al*. [57] used a combination of CFD and FSI to determine how the fluid flow influenced the mechanical stimulation of the cells inside the scaffold. Additionally, this study also used the FEM to determine how mechanical compression affected the osteoblast cells. The results showed that while the fluid flow stimulated the bridged cells within the scaffolds, there was almost no stimulation in the attached cells. The mechanical compression tests demonstrated the opposite effect, with the attached cells experiencing much higher stimulation than the bridged cells. The paper suggests that a combination of flow perfusion and mechanical compression might be the optimal method to obtain the required stimulation for both bridged and attached cells.

### (b) Experimental validation of CFD

Most of the studies discussed in this review develop their numerical simulations by using CFD parameters taken from the literature (such as inlet velocity and fluid viscosity), or by validating their new numerical models by comparing the results with values from existing research. However, some papers validated their numerical results by comparing the computational results with values obtained through experimental testing [24,25,43,47,56,61].

Montazerian *et al*. [43] and Zhianmanesh *et al*. [47] both conducted constant head permeability tests to determine the correction factor between the numerical and experimental scaffold permeability. Montazerian *et al*. [43] conducted this experimental test for seven different scaffold designs and with five different fluid heights. They determined that the correction factor between their numerical and experimental results was between 0.062 and 0.145 (for an experimental fluid-height range of 50–10 mm, respectively). Zhianmanesh *et al*. [47] conducted their experimental tests for 12 different scaffold designs and with five different fluid heights, repeating the test three times for each configuration. They determined that the correction factor between their numerical and experimental results was between 0.12 and 0.20, for fluid heights between 20 and 60 mm. The discrepancies between numerical and experimental results were attributed to differences between the computational and three-dimensional printed models, as well as not having considered the surface roughness of the scaffold wall on the numerical models.

In a similar permeability study, Yu *et al*. [56] used a falling head permeability test to validate the numerical results. Once again, that study found that the computational results are much higher than the experimental ones; however, the two sets of results show a *R*^2^ > 0.99, demonstrating the reliability of the CFD models. Entezari *et al*. [61] also implemented an experimental permeability test, more specifically a peristaltic pump permeability test, to validate the results that were obtained numerically. Two different scaffold geometries were tested with six scaffolds for each geometry, to obtain statistically reliable results. The experimental values of the pressure difference (and consequently of the scaffold permeability) were very close to the numerical ones, highlighting the effectiveness of numerical simulations.

Finally, Campos Marin *et al*. [24] also implemented experimental validation of their computational results. They analysed cell seeding efficiency using CFD analysis for the numerical component and a DNA assay for the experimental component. For each fluid flow configuration, five scaffolds were tested experimentally, whereas only one computational simulation was carried out for each configuration. They found a good agreement between the *in silico* and *in vitro* cell seeding efficiencies, even though this value was 35% higher in the numerical simulations. This difference was likely due to the limitation of the computational model to simulate realistic cell adhesion events or formation of cell clusters. Another work also used experimental results to validate its numerical results [25]. That paper compared the results of its CFD models with equivalent particle-tracking velocimetry (PTV) experiments. The authors found that the CFD results agreed with the PTV experiments on the fact that cells followed the fluid streamlines due to the strong effect of fluid drag.

### (c) Scaffold optimization

**Figure 6.**
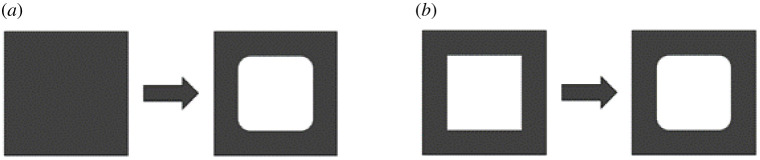
Types of possible scaffold optimization: (*a*) topology optimization and (*b*) shape optimization.

A BTE scaffold is defined by a multitude of parameters, which include geometry, wall thickness, porosity and the manufacturing material. These parameters can be optimized to reach a certain goal, such as obtaining a scaffold with a pre-established compressive strength. This is known as an optimization process and, although there are several different optimization algorithms, most of them follow the same framework [69]. The process starts with an initial step which defines an initial geometry; the material properties of the structure; the constraints and the chosen objective function for that specific optimization process. Afterwards, a numerical component is implemented to analyse the relevant properties of the initial geometry. If the resulting properties do not reach the objective, then an optimization function is used to obtain a new geometry, according to the predefined constraints. This process repeats itself iteratively until a new structure satisfies the objective function. Optimization can be divided into optimization and shape optimization [14] (figure 6).

However, these optimization methods, even though they present an appealing tool to be used alongside CFD in scaffold design, are almost always exclusively used for optimizing the scaffolds' mechanical properties [18,19,21,70]. In recent years, very few studies have combined optimization and a CFD analysis. One exception is the paper by Entezari *et al*. [61] that used a combination of CFD analysis and the FEM to optimize lattice scaffolds in terms of their permeability and compressive strength. To address the competing objectives of maximizing permeability and maximizing compressive strength, a multi-objective particle swarm optimization algorithm coded in Matlab was chosen. However, no other study was found that attempted to optimize other fluidic properties, such as WSS.

## 5. Conclusion

CFD analysis is more and more a crucial tool in BTE, as it allows tailoring and evaluating the fluidic properties of several different scaffold structures. Consequently, this numerical method allows the study of the behaviour of the cells that will populate the inner space of the scaffolds. The papers discussed in this review have demonstrated how changes to the material, manufacturing process or geometry of a given scaffold may significantly influence its properties. In addition, these studies have demonstrated how the porosity of a scaffold is fundamental in determining its fluidic properties: more porous scaffolds are more permeable but have overall lower WSS. For scaffold designed for *in vivo* applications, the combination of CFD with FEM analyses has highlighted how the fluidic properties of a given scaffold are not separated from its mechanical properties. Several studies have concluded how increasing the porosity of a scaffold would increase its permeability, but this would come at the expense of its mechanical properties, namely its compressive strength and Young's modulus. This conclusion emphasizes the need for *in vivo* BTE scaffolds to take into consideration both sides of their design: providing the bone with the required mechanical support and possessing a microenvironment conducive to cellular attachment, differentiation and growth. Towards achieving this goal, a balance must be reached between mechanical and fluidic properties of the structure.

Studies available in the literature have already employed CFD analyses in conjunction with cellular growth algorithms to obtain a better understanding of the cellular behaviour inside the scaffolds. These studies revealed that fluid flow rate had a major impact in the WSS experienced by the cells, which in turn influenced their growth and differentiation.

It seems very few papers have attempted to implement a CFD analysis as a component of an optimization algorithm, in order to optimize the fluidic properties of scaffolds, with the exception of the previously discussed paper by Entezari *et al*. [61]. Most of the recent BTE scaffold optimization studies focused on FEM with emphasis on mechanical properties. This raises an interesting question on how to design an optimization process for scaffold design that could account for both fluidic and mechanical properties, i.e. how would the objective function of such a process be implemented, given the opposite nature of maximizing a scaffold's permeability and mechanical properties?

A major limitation of the current CFD studies is that a majority of reviewed papers did not present a specific purpose (for specific bones) other than a generic application in BTE. The only guidelines offered were to use the scaffolds for *in vitro* bioreactors or as a component in spinal interbody fusion cages [51]. As different BTE applications have different requirements in terms of their scaffold characteristics, not defining said requirements could severally limit the relevance of the studies. Additionally, another challenge faced by current CFD studies is the (high) discrepancy between the numerical and experimental results [43,47,56]. This difference, which is probably caused by not taking into account certain factors (such as surface roughness) in the CFD simulations, may inhibit the ability to accurately determine the characteristics of a given scaffold design.

In summary, future research into the properties of BTE scaffolds needs to consider the relation between the mechanical and fluidic properties of scaffolds, with improvements to one usually negatively affecting the other. The present study also highlighted the lack of research into the application of CFD analyses for the optimization of BTE scaffolds. This review underlines that there are still no wide-range studies employing optimization to simultaneously improve the permeability, WSS and compressive strength of bone-replacement scaffolds.

## Data accessibility

This article has no additional data.
